# Current-Voltage and Transport Characteristics of Heterogeneous Ion-Exchange Membranes in Electrodialysis of Solutions Containing a Heterocyclic Amino Acid and a Strong Electrolyte

**DOI:** 10.3390/membranes13010098

**Published:** 2023-01-12

**Authors:** Tatiana Eliseeva, Anastasiia Kharina

**Affiliations:** Department of Analytical Chemistry, Voronezh State University, 1 Universitetskaya pl., 394018 Voronezh, Russia

**Keywords:** heterocyclic amino acid, transport, ion-exchange membrane, electrodialysis, current-voltage curve, fouling

## Abstract

The alterations in current-voltage and transport characteristics of highly basic and strongly acidic ion-exchange membranes, during the electrodialysis of solutions containing a heterocyclic amino acid and a strong electrolyte, were studied. An increase in the catalytic activity of the water splitting process at the surface of heterogeneous MK-40 and MA-41 membranes upon prolonged contact with proline and tryptophan solutions was found. A significant effect of electroconvection on the components mass transfer through the cation-exchange membrane in the intensive current mode of electrodialysis was revealed for the solution containing a heterocyclic amino acid along with mineral salt (NaCl). This led to a reduction in the length of the “plateau” of the membrane’s current-voltage characteristics, in comparison with the characteristics for an individual sodium chloride solution with the same concentration. The changes in the characteristics of the studied ion-exchange membranes caused by contact with solutions containing heterocyclic amino acids during electrodialysis were reversible when applying electrochemical regeneration (cleaning in place) using the overlimiting current mode, corresponding to the region of facilitated transport for these ampholytes.

## 1. Introduction

Amino acids are often required as high-purity compounds in medical, pharmaceutical, and cosmetic industry settings; however, when they are produced by the conventional methods, such as microbiological, chemical, or enzymatic synthesis, the target product is contaminated with impurities, in particular mineral salts. The modern electromembrane method–electrodialysis solves the problem of separating amino acids from mineral salts [1,2,3,4,5,6,7,8].

The use of electrodialysis for the demineralization of amino acid solutions requires the development of optimal conditions for extraction of a high-purity product with minimal losses. Knowledge of the features of amino acid mass transfer through ion-exchange membranes, depending on the pH of the solution and on the applied electric potential gradient, makes it possible to control the direction and magnitude of the mass fluxes in electrodialysis. There are some common features of amino acid transport [9,10,11] and some special features resulting from the side radical size, charge, structure, and hydration [12].

The properties of the side radical should be taken into account when purifying solutions of various amino acids in an electromembrane system.

However, studies of the transport and current-voltage, structural, and other characteristics of ion-exchange membranes in the electrodialysis of solutions containing a heterocyclic amino acid are represented by only a few works [13,14,15].

According to modern concepts, the demineralization of solutions in intensive current regimes through electrodialisys (ED) is a promising approach, since it entails the achievement of a high degree of desalination. In overlimiting current conditions, the fluxes of solution components through the ion-exchange membranes are associated with the transport of hydrogen and hydroxyl ions generated at the membrane–solution interface in the course of water splitting and are also determined by the influence of the phenomena of limiting the current exaltation, gravitational convection, and electroconvection [16,17], which lead to a complex mechanism of component mass transfer. The non-linear dependence of amino acid fluxes through the membranes on the current density is additionally determined by the barrier effect and the effect of facilitated electromigration [9,10,11]. Descriptions of mathematical models can be found in the scientific literature that allow predicting the efficiency parameters of solution desalination for various designs of electromembrane systems at current density values below the limiting one, as well as in the overlimiting mode [17,18,19].

However, mathematical models do not take into account the complex nature of the dependence of ampholyte transport on current density in ED, as well as a number of undesirable side processes caused by the specific interactions of solution components. An undesirable phenomenon that can accompany electromembrane demineralization of amino acid solutions, in particular of heterocyclic type, is organic fouling of the ion-exchange membranes, affecting their performance efficiency and even their service life.

Fouling is a drawback of ED, as well as of other membrane technologies. It results in an increase of membrane resistivity, typically causing a decrease of flux through a membrane and increased energy consumption. Fouling is often a severe problem for ion-exchange membranes. Different types of agents can act as foulants [20,21,22]. The mechanisms of the inorganic, organic, and biological fouling occurring in the treatment of natural and waste water have been discussed in the literature [23,24,25,26]. Precipitation, deposition of colloidal particles, electrostatic and hydrophobic interactions, and formation of coordination compounds, as well as biological processes, are indicated among the predominant mechanisms of fouling for ion-exchange membranes. In the literature, fouling is classified as reversible or irreversible [25,27].

A number of researchers exclusively gave attention to issues of ion-exchange membrane fouling by organic substances in electrodialysis [22,28,29,30,31].

Various methods are applied to studying the fouling phenomenon for ion-exchange membranes. Many procedures for estimating fouling, as well as the transport, mass-transfer, and electrochemical characteristics of fouled membranes, have been suggested. A thoughtful systematization of useful techniques was published recently [23].

Fouling of an ion-exchange membrane affects its electrochemical characteristics; and their control is an integral part of assessing the performance properties of a whole system. Previous papers have considered changes in the current-voltage characteristics and conductive properties of ion-exchange membranes in various ampholyte-containing solutions [32,33].

It should be noted that the fouling phenomenon in electrodialysis leads to a decrease of membrane conductivity, due to the formation of a surface fouling layer or internal (bulk) membrane fouling, with blocking of membrane ion-exchange groups by fouling agents [34,35].

Surface and/or bulk fouling leads to certain changes in the shape of current-voltage curves. For example, changes have been shown in the slope of the ohmic region of the current-voltage curve of fouled membranes in comparison with pristine membranes. Changes in the limiting current density and plateau length values have been found. It is known that fouling can reduce the conducting phase fraction of membranes [36,37,38].

The authors of [39] showed changes in the impedance spectra of fouled membranes, a decrease in the resistance to charge transfer, and an increase in the capacitance of the double layer.

Zeta potential measurements can provide valuable information about the effect of the electrostatic forces acting between fouling agents and membrane surfaces [34,40].

Contact angle measurements allow estimating surface hydrophobicity changes due to fouling [34,41].

The method of IR spectroscopy makes it possible to establish changes in the structure of the ion-exchange materials used in the electrodialysis of different organic foulants solutions; for example, water solutions containing humate, bovine serum albumin, or sodium dodecylbenzenesulfonate [34], as well as nanofiltrated acid whey solution [42].

Another method that is widely used for the detection of ion-exchange membrane surface fouling is electron microscopy [40,42,43].

The tendency of ion-exchange membranes to be fouled by organic ampholytes, such as heterocyclic amino acids, requires finding out the cause of the fouling and developing methods to prevent/eliminate it. Due to a lack of knowledge about the electrochemical properties of membranes used in the demineralization of such amino acids solutions, the focus of this work was to study changes in the mass transport and current-voltage characteristics of heterogeneous ion-exchange membranes in the prolonged electrodialysis of a heterocyclic amino acid and a strong electrolyte mixed solution.

## 2. Materials and Methods

### 2.1. Materials

L-optical isomers of heterocyclic amino acids tryptophan and proline (Sigma-Aldrich, Burlington, MA, USA) were used in the experiments.

Tryptophan (Trp) is an essential heterocyclic amino acid. It belongs to a number of hydrophobic amino acids. Trp has an aromatic nucleus of indole, which can participate in specific interactions.

Proline (Pro) is also a heterocyclic amino acid, but it is not aromatic like tryptophan, and a single nitrogen atom in its structure is part of the imino-group.

The presence of both an amino (imino) group and a carboxyl group in the structure of amino acids leads to the fact that their chemical properties and physicochemical behavior as ampholytes strongly depend on the pH of the medium; they can exist in a solution as cations, anions, and zwitterions.

Some characteristics of the studied amino acids are presented in Table 1.

Heterogeneous anion-exchange membranes (AEM) MA-41 and cation-exchange membranes (CEM) MK-40 produced by LLC UCC “Shchekinoazot”, Pervomaisky, Russia (Table 2) were used. The AEM is composed of a styrene–divinylbenzene copolymer with functional quaternary ammonium groups. The CEM is composed of a styrene–divinylbenzene copolymer with functional sulpho groups. Both membranes contain polyethylene as an inert binder in their structure.

### 2.2. Methods

**Figure 1:**
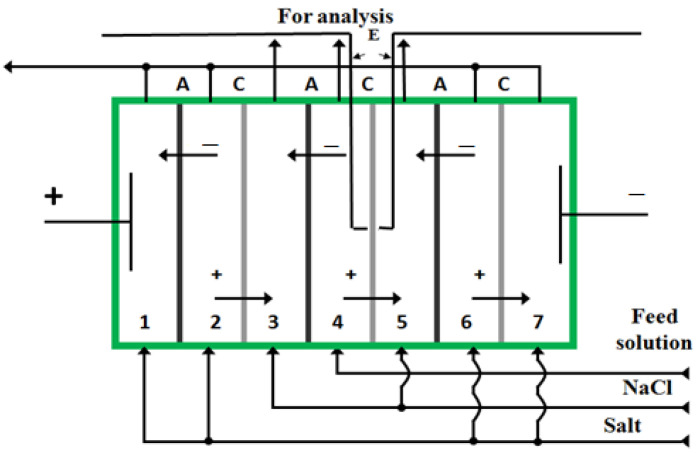
Seven-compartment electrodialysis cell with alternating cation (C)- and anion (A)-exchange membranes, “+”—cation, “−”—anion, E—electrodes (Ag/AgCl).

The electrodialysis cell used had seven compartments separated by alternating cation-exchange and anion-exchange membranes. The distance between the membranes was 10 mm. Channel height was 20 cm. The linear flow rate of solutions was 0.1 cm·s^−1^. Electrodialysis was carried out in galvanostatic mode. The anode material was platinum; the cathode was made of stainless steel. The concentration of the salt solution (K_2_SO_4_, ZAO Vekton, Saint Petersburg, Russia) supplied to compartments 1, 2, 6, and 7 was 10 times higher than the concentration of the solution supplied to compartments 3, 4, and 5. As a result, the limiting current density values for the cation-exchange and anion-exchange membranes separating compartments 4 and 5, and also compartments 4 and 3, were lower compared to the values at which the limiting state was reached for the membranes separating sections 1 and 2, 2 and 3, 5 and 6, and 6 and 7. In compartments 1, 2, 6, and 7, a solution of potassium sulfate was supplied; in compartments 3 and 5, this was a solution of sodium chloride (ZAO Vekton, Saint Petersburg, Russia). A mixed solution of sodium chloride and amino acid was fed into compartment 4 of the electrodialyzer. The feed concentration of mineral salt for compartments 3, 4, and 5 was 0.01 M. The feed concentration of amino acid was 0.02 M. Analysis was carried out of the effluents of compartments 3, 4, and 5. Silver chloride electrodes were placed on both sides of the membrane under study, at a distance of 0.2 mm, to record the current-voltage characteristics (CVC). A scheme of the electrodialysis cell is shown in Figure 1.

The pH value of the mixed solutions being fed into the 4th compartment was in the range 5.7–6.5, which is close to the isoelectric point of tryptophan or proline. The pH value of solutions was measured using a laboratory ionometer I-160MI (RPA Measuring equipment, Moscow, Russia).

The concentration of tryptophan in the samples was measured using spectrophotometry [45]; proline was analyzed with the photometric method, and the procedure was based on the formation of a blue-colored amino acid complex with Cu^2+^-ions [46].

The content of Cl^−^-ions was detected by precipitation titration, using K_2_CrO_4_ as an indicator [47]. The quantitative analysis of sodium ions was accomplished using flame photometry [48].

Cleaning of the ion-exchange membranes was carried out with an electrochemical procedure in overlimiting mode, using the range of facilitated electromigration effect [11].

The component flux through the membrane was calculated according to Equation (1) [16]:

(1) $$J=\frac{C\cdot V}{\tau \cdot S}\;$$

where *J*—flux of a component through the ion-exchange membrane, mol·cm^−2^·s^−1^;

*C*—concentration of the solution, mol·dm^−3^; *V*—volume, dm^3^; *τ*—sampling time, s; and *S*—working area of the ion-exchange membrane, cm^2^.

The effective transport numbers for hydroxyl ions were measured using an electro-analytical procedure during electrodialysis and calculated according to Equation (2) [16]:

(2) $$T=\frac{z\cdot F\cdot J}{i}$$

where *T*—effective transport number; *F*—Faraday constant, C·mol^−1^; *z*—ion charge; *i*—current density, A·cm^−2^; and *J*—flux of OH^−^ -ions, mol·cm^−2^·s^−1^.

In this work, the changes in the state of the ion-exchange membrane surface during the electrodialysis of solutions containing heterocyclic amino acids were studied with atomic force microscopy, using a Solver P47 Pro scanning probe microscope (Ltd NT-MDT Moscow, Russia). Preliminarily dried samples of ion-exchange materials at 50 °C were scanned with an NSG20 cantilever, to determine the surface topography. The semi-contact method was used. The experiments were carried out in air medium at a temperature of 25 ± 1 °C. The sensitivity of the probe and the scanner made it possible to obtain images of the surface with a high vertical resolution of at least 1 nm and up to 40 nm horizontally. The measurement results obtained using a probe microscope were two-dimensional and three-dimensional digital images of the surface. Processing of the microphotographs was based on the analysis of the standard arithmetic mean of the surface parameters using FemtoScanOnline software (femtoscan.2.4.25, 42.7 Mb) [49].

## 3. Results and Discussion

Amino acids adsorption on the surface and non-exchange sorption in the bulk of the membrane phase in contact with treated solutions are undesirable phenomena in the desalination of solutions through electromembrane methods. These phenomena can be caused by various types of physical and/or chemical interactions of the amino acid with the ion-exchange membrane functional groups and matrix. To unravel the effect of such interactions leading to fouling, the current-voltage and transport characteristics of heterogeneous membranes that had been in prolonged contact with solutions containing a heterocyclic amino acid and a strong electrolyte in ED were studied.

### 3.1. Current-Voltage Characteristics of Ion-Exchange Membranes in Electrodialysis of a Solution of Heterocyclic Amino Acid–Mineral Salt

Changes in the CVC of MA-41 and MK-40 membranes were detected during electrodialysis of model solutions containing tryptophan (or proline) mixed with sodium chloride. The continuous contact time of the membrane with a solution containing a heterocyclic amino acid in the electromembrane system was 36 h. The CVCs of the ion-exchange membranes had a classical shape with three regions. The first corresponded to the formation of a diffusion boundary layer. As the concentration gradient increased, the membrane voltage increased. In this case, the dependence of the current density (i) on the membrane voltage was linear. Then, an exponential approximation of i to the limiting current density (i_lim_) was observed. When reaching i_lim_, water splitting occurred, which was catalyzed by the functional groups of the ion-exchange membranes. In the intensive current mode, at higher values of current density, the membrane potential again increased linearly.

The presence of proline or tryptophan with a concentration of 0.02 M in a mineral salt solution did not affect the i_lim_ value observed for cation-exchange and the anion-exchange membranes, when the fraction of amino acid cations and anions in the solution was minimal and the main part of the organic ampholyte was in the form of bipolar ions.

However, with the presence of an amino acid in the system, the “plateau” length of the current-voltage curve for the MK-40 heterogeneous membrane shortened, and its slope increased. This could have been be caused by several factors. In intensive current mode, the appearance of hydroxonium ions in the cation-exchange membrane phase, as a result of water splitting at the interphase boundary, leads to the recharging of amino acid bipolar ions into cations. Amino acid cations become additional current carriers in the system (the effect of facilitated migration).

**Figure 2:**
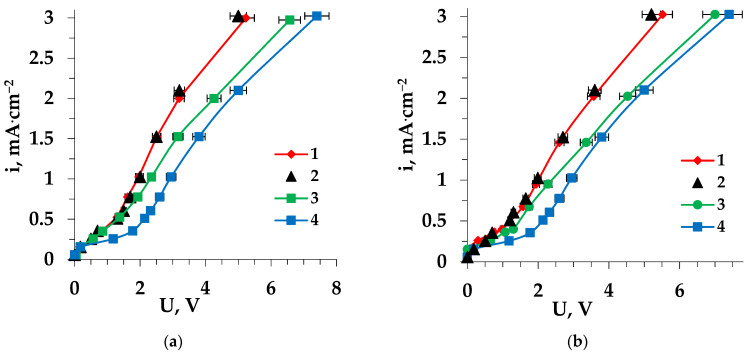
Current-voltage characteristics of the MK-40 cation-exchange membrane in the electrodialysis of Trp + NaCl (**a**) and Pro + NaCl (**b**) solutions: 1—pristine membrane, 2—regenerated membrane, 3—fouled membrane; 4—pristine membrane in the ED of NaCl solution.

In addition, due to the hydrophobic properties of the side radicals of sorbed heterocyclic amino acids [50], the degree of hydrophilicity of the cation-exchange membrane surface decreases [51]. This causes an increase of the contribution of electroconvection to the mass transfer through a membrane, which results in a reduction of the “plateau” length and an increase in its slope on the CVC. The differences in the hydrophobicity and structure of amino acids such as Trp and Pro affect the electrochemical and transport characteristics of membranes and lead to some features of demineralization of solutions by electrodialysis. In accordance with the different hydrophobicities of the considered amino acids sorbed on the membrane surface (Pro < Trp [52]), the degree of hydrophilicity decreased and, accordingly, the contribution of electroconvection increased. For the electrodialysis of the solution containing Pro, the contribution of electroconvection was lower than in the system with Trp. As a result, the reduction in the length of the CVC “plateau” of the cation-exchange membrane in the Pro + NaCl solution was less than in the Trp + NaCl solution. Figure 2 shows the CVCs of the pristine MK-40 membrane in the ED of solutions of the studied amino acids mixed with sodium chloride (1), in comparison with the fouled membrane after prolonged performance (36 h) in this solution (3), and for the membrane after electrochemical regeneration (2). The CVC of the pristine membrane in the ED of sodium chloride solution (4) is also presented.

Confirmation of an increase in the hydrophobicity of the ion-exchange membrane surface in contact with the solution containing a mineral salt and a heterocyclic amino acid, compared with the individual solution of a mineral salt, is presented in [14]. An increase in the water contact angle for the membranes operating in solutions containing heterocyclic amino acids was revealed.

**Figure 3:**
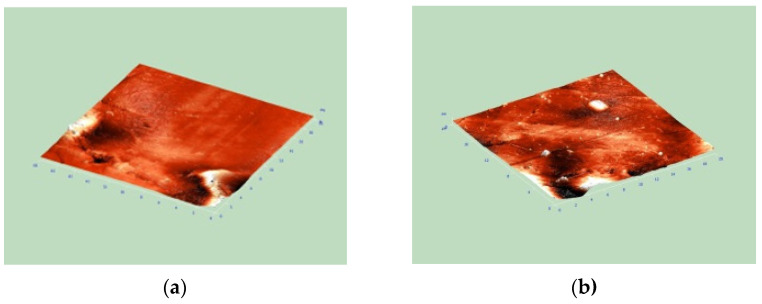

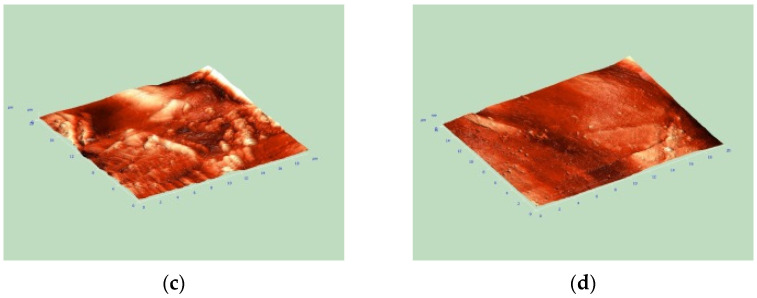
Three-dimensional images of the surface of the MA-41 membrane: (**a**) OH^−^-form, (**b**) Pro- form, (**c**) membrane after prolonged contact with solution containing Pro during electrodialysis, (**d**) membrane after regeneration (Scanned field—(20 × 20) × 1.6 μm).

Changes in the geometric parameters of the surface of the ion-exchange membranes that were in contact with solutions containing heterocyclic amino acid during electrodialysis were found in this work. With an increase in the contact time, an increase in the roughness of the membrane surface was detected. The calculated roughness parameters for the MA-41 and MK-40 membranes are shown in Table 3. As an example, Figure 3 shows images for the pristine, fouled, and regenerated MA-41 membrane.

There was an increase in the geometric inhomogeneity of the membrane surface, as well as the blocking of its pores. This occurred as a result of the ion-exchange, as well as the non-exchange sorption of a heterocyclic amino acid due to weak ion-dipole and dipole-dipole interactions; and in the case of tryptophan, additionally, hydrophobic interactions of aromatic fragments of the amino acid structure and membrane [4,53]. An increase in the surface roughness of membranes that had been in contact with solutions of heterocyclic amino acids may also have been the cause of the increase in the contribution of electroconvection to the mass transfer of components through the membrane.

Regeneration in the intensive current mode of ED ensured the restoration of the membrane surface morphology.

An increase in the length of the CVC “plateau” was observed for the MK-40 membrane during the electrodialysis of solutions containing heterocyclic amino acid and sodium chloride for 36 h in comparison with the pristine membrane (Figure 2). This was due to the accumulation of sorbed amino acid with carboxylic, imino, and/or amino groups in the membrane phase, which led to an increase in the catalytic activity of the membrane with respect to the water splitting reaction. The similar catalytic effects of certain organic substances, in particular surfactants, were noted in [54,55]. Moreover, the revealed change in the CVC of the membrane may have been associated with an increase in the electrical resistance of the membrane due to the uptake of amino acid.

**Figure 4:**
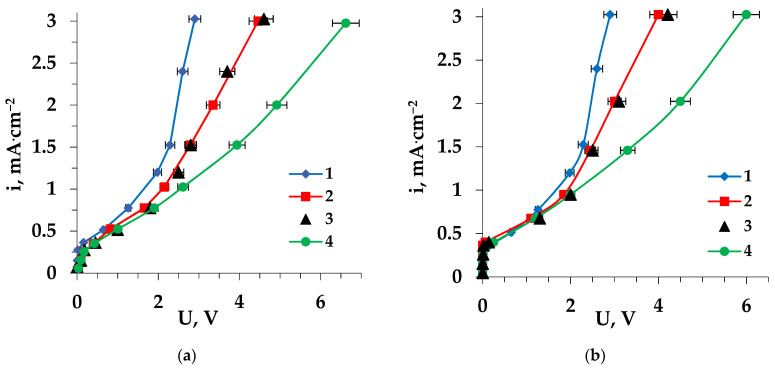
Current-voltage characteristics of the MA-41 anion-exchange membrane during the electrodialysis of Trp + NaCl (**a**), Pro + NaCl (**b**) solutions: 1—pristine membrane during the ED of NaCl solution, 2—pristine membrane, 3—electrochemically-regenerated membrane, 4—fouled membrane.

The electrochemical behavior of the anion-exchange membrane was also considered. Figure 4 shows current-voltage curves for the MA-41 membrane, which were registered during the electrodialysis of all the studied solutions. The voltage of the pristine and fouled anion-exchange membrane in Trp + NaCl and Pro + NaCl solutions was higher than in NaCl solution at the same values of current density.

The presence of amino acid (tryptophan or proline) in the feed solution led to an increase in the length of the plateau, corresponding to the i_lim_ in the CVC of the membrane. The low mobility of tryptophan and proline, along with the possibility of hydrophobic intermolecular interactions of tryptophan with the membrane matrix, reduced the conductive properties of the membrane.

In addition, the shape of the CVC of the anion-exchange membrane was affected by the structure of the specific side radical of sorbed amino acid. The catalytic activity of the N–H group of the Trp indole ring and N–H group of the Pro pyrrole ring was higher than that of the quaternary ammonium base of the MA-41 membrane [54].

Therefore, the more intensive water splitting at the interface boundary of the anion-exchange membrane–solution suppressed the electroconvection, which led to an increase in the CVC “plateau” of the membrane in the solutions with Trp and Pro.

In solutions containing these heterocyclic amino acids (Trp or Pro), after prolonged contact (36 h) during ED, an additional increase in the length of the CVC “plateau” of the MA-41 anion-exchange membrane was detected (Figure 4).

**Figure 5:**
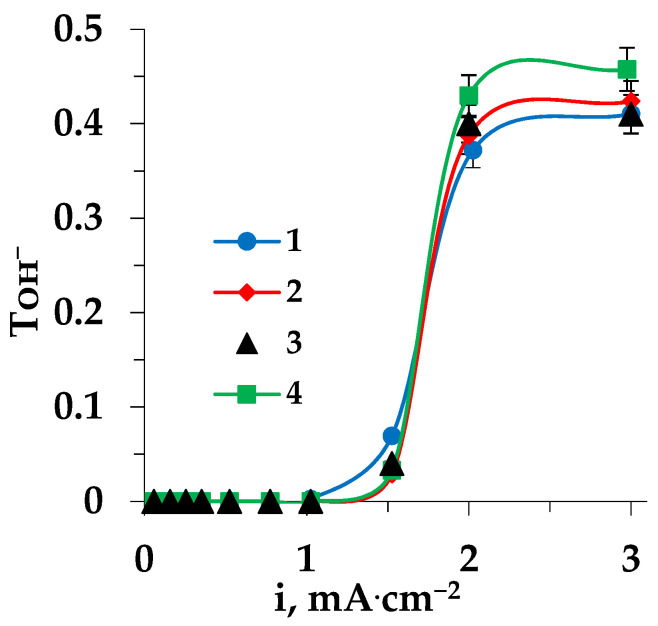
Dependence of the hydroxyl ion effective transport number on the current density for the MA-41 membrane during the electrodialysis of Trp + NaCl solutions: 1—pristine membrane in ED of NaCl solution, 2—pristine membrane, 3—regenerated membrane, 4—fouled membrane.

The increase in the catalytic activity of the MA-41 anion-exchange membrane, observed upon prolonged contact with a heterocyclic amino acid solution during ED, was confirmed by the higher values of hydroxyl ion effective transport. Figure 5 shows the dependence of the effective transport number on the current density during the electrodialysis of the Trp-containing solution.

It should be noted that the presence of not only the N–H group of the indole ring in tryptophan, but also the presence of the primary amino group, led to the highest values for the effective transport of hydroxyl ions, even higher than in the case of the electrodialysis of the sodium chloride solution.

Carrying out electrochemical regeneration of ion-exchange membranes (with some kind of cleaning in place) made it possible to restore the shape of their current-voltage characteristics to the state of the pristine membrane. The effective transport numbers of hydroxyl ions in the intensive current mode for the regenerated membrane also returned to the values for the pristine membrane (Figure 5, curve 3).

The IR spectra of the anion-exchange material regenerated after long-term performance in the tryptophan-containing solution and of the pristine sample were approximately the same [56]. All alterations in the IR spectrum of the fouled membrane in comparison with the pristine membrane (absorption bands at 1577 cm^−1^, 1109 cm^−1^, corresponding to the vibrations of the COO^−^ anion of amino acid, at 1068 cm^−1^, indicating the appearance of primary amino groups in the anion-exchange material) disappeared in the IR spectrum of the regenerated membrane. This indicated the reversibility of the fouling.

### 3.2. Transport Characteristics of the Ion-Exchange Membranes during the Electrodialysis of a Heterocyclic Amino Acid–Mineral Salt Solution

The effects of the proline and tryptophan fluxes through the ion-exchange membranes on the current density in the electrodialysis of a mixed solution with a mineral salt were studied.

**Figure 6:**
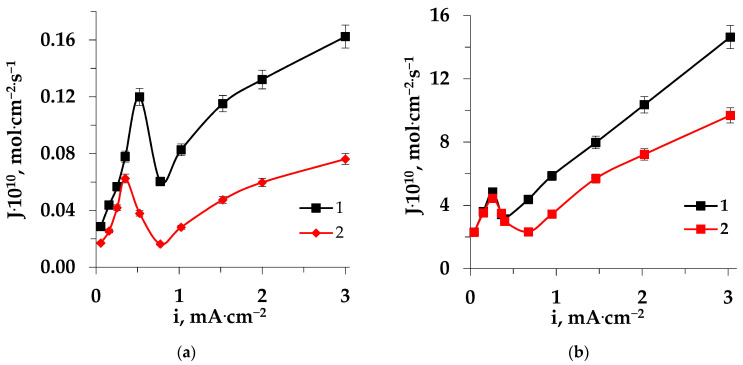
Dependences of the amino acid fluxes through the membranes on the current density during electrodialysis of Trp + NaCl (**a**) and Pro + NaCl (**b**) solutions: 1–MK-40, 2–MA-41.

The fluxes of the heterocyclic amino acids through the MK-40 cation-exchange membrane reached higher values than through the MA-41 anion-exchange membrane (Figure 6). The difference in amino acids fluxes through these membranes could be explained, in particular, by the higher capacity of the MK-40 cation-exchange membrane (in comparison with MA-41) for amino acids [14] (and for the mineral ions [44]). The maximum of the dependences corresponded to the limiting current density. After reaching the limiting current density, a decrease in amino acid fluxes was observed, due to the action of the barrier effect [9]. In the intensive current mode, an increase in amino acid fluxes with an increase in current density was revealed, due to the effect of facilitated electromigration [11]. The barrier effect and the effect of facilitated electromigration determined the main features of the mass transfer of organic zwitterlites. A brief interpretation of the dependences of the amino acid flux–current density is given below.

An increase in the applied gradient of the electric potential was accompanied by a linear increase in organic ampholyte fluxes at low values of current density. The increase of the fluxes under limiting mode can be explained by the conjugated transport of amino acid with mineral ions and by the migration of amino acid monopolar ions, which were present in solution in small quantities, even at the isoelectric point [9]. When the limiting current density was reached, the concentration of ions near the membrane surface involved in the mass transfer became critically low. The resistance near the membrane surface increased and the water dissociation reaction proceeded at the membrane–solution interface, leading to the generation of additional current carriers–hydroxyl and hydroxonium ions. There was an increase in the content of hydroxyl ions at the interface of the cation-exchange membrane (impermeable to negatively charged ions)–solution and of hydroxonium ions at the interface of the anion-exchange membrane, which was selective to negatively charged ions. A change in pH near the membrane surface served as a barrier to the transport of the corresponding ampholyte ions through the cation- and anion-exchange membranes, reducing the amino acid fluxes. H_3_O^+^ and OH^−^ ions formed in the course water splitting took part in the transfer of electric current through the cation- and anion-exchange membranes, along with ions of mineral salt [9,11].

In the more intensive current mode (i = 1.6–2.8 i_lim_ for various systems membrane/solution), the action of the barrier effect was restricted by various convection mechanisms, causing mixing of the solution and the destruction of the diffusion boundary layers with pH gradients in the dilute compartment. The effect of facilitated electromigration was seen. The conjugated transport of amino acids and ions formed during the process of water splitting, with the formation of cations and anions from bipolar amino acid ions occurred, which is called facilitated electromigration. Under the overlimiting conditions of electrodialysis, the progress of electroconvection, gravitational convection, exo-effects, and exaltation effects also influenced the magnitude of the component fluxes through the ion-exchange membranes [17].

The difference in the structure of the amino acids under consideration was the reason for the peculiarities of their mass transfer through both the cation-exchange and anion-exchange membranes. The lower mobility of tryptophan ions in comparison with proline ions led to the minimum values of the fluxes through both the MK-40 and MA-41 membranes.

**Figure 7:**
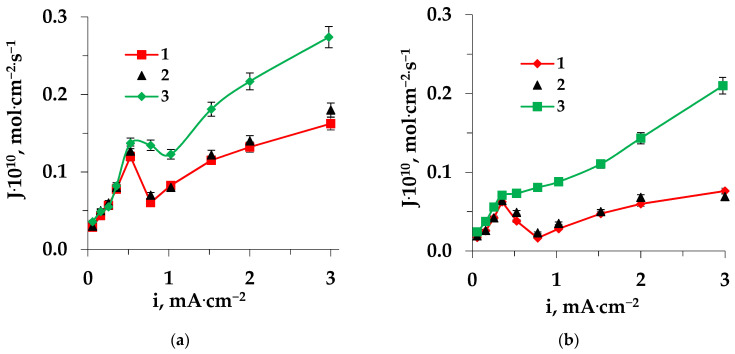
Dependences of amino acid fluxes through the MK-40 (**a**) and MA-41 (**b**) membranes on the current density during electrodialysis of Trp + NaCl solution: 1—pristine membrane, 2—regenerated membrane, and 3—fouled membrane.

**Figure 8:**
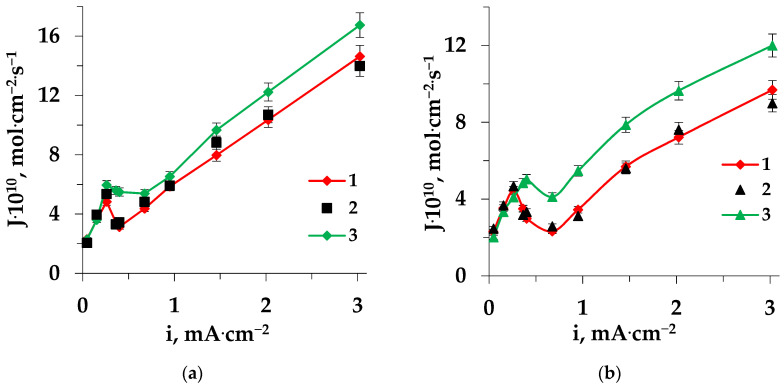
Dependences of amino acid fluxes through the MK-40 (**a**) and MA-41 (**b**) membranes on the current density during electrodialysis of Pro + NaCl solution: 1—pristine membrane, 2—regenerated membrane, and 3—fouled membrane.

An increase in amino acid mass transfer through both the cation-exchange and anion-exchange membranes after prolonged contact (36 h) with a solution containing a heterocyclic amino acid during ED (Figure 7 and Figure 8) was associated with intensive electroconvective transport of amino acids, enhanced by an increase in surface roughness and hydrophobicity, and also with conjugated transport with ions formed in the process of the catalyzed reaction of water splitting at the interphase boundary.

With prolonged use of the membranes, a decrease in the influence of the barrier effect was observed, apparently due to the accumulation of amino acids in the membrane phase and on the surface, leading to an increase of the impact of electroconvection and an increase in the effect of facilitated transport.

**Figure 9:**
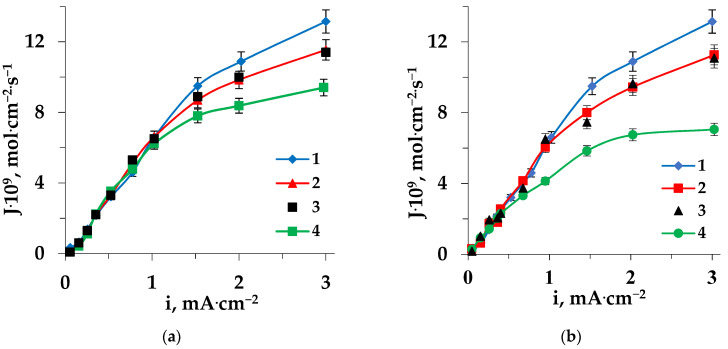
Dependences of chloride ions fluxes through the MA-41 membrane on the current density during electrodialysis of the solutions Trp + NaCl (**a**), Pro + NaCl (**b**): 1—pristine membrane during ED of NaCl solution, 2—pristine membrane, 3—regenerated membrane, 4—fouled membrane.

The decrease in the fluxes of chloride ions through the MA-41 membrane that had been in contact with a solution containing a heterocyclic amino acid for a long time (Figure 9) was due to an increase of the competitive transport of amino acids in the region of facilitated transport.

After electrochemical regeneration of the ion-exchange membranes, amino acid fluxes through the MK-40 and MA-41 membranes returned to their original values for the pristine samples.

The study of changes in electrochemical and transport characteristics of ion-exchange membranes showed an undesirable phenomenon of organic fouling, which worsened the efficiency of the desalination of the heterocyclic amino acid solutions. However, in contrast to the long-term operation of ion-exchange membranes, when the functional groups of quaternary ammonium bases were irreversibly transformed into secondary and tertiary ones (ageing phenomenon) [41,57,58], the “organic fouling” in the studied solutions was reversible under electrodialysis conditions and could be reduced using the intensive current mode. Membranes can be regenerated electrochemically (overlimiting current density, corresponding to the influence of the effect of facilitated electromigration), and this can ensure the process of effective demineralization of amino acid solutions through electrodialysis.

## 4. Conclusions

The functional groups of tryptophan (N-H of the indole cycle, as well as carboxyl and primary amino groups) or proline ((N-H of the pyrrole cycle), accumulated at the membrane surface, upon prolonged contact with a solution containing one of these amino acids, provided an increase in catalytic activity with respect to the water dissociation reaction of MA-41 membranes having highly basic quaternary amino groups and MK-40 membranes having sulfo groups. As a result, an increase in the length of the “plateau” of the current-voltage characteristics of the ion-exchange MK-40 and MA-41 membranes was observed.

The significant influence of electroconvection on the mass transfer through the cation-exchange membrane during the intensive current mode electrodialysis of the solution (heterocyclic amino acid–sodium chloride) led to a reduction in the “plateau” length of the current-voltage characteristics of the membrane compared to an individual solution of sodium chloride. The higher hydrophobicity of tryptophan in comparison with proline provided a greater reduction in the length of the “plateau” of the current-voltage characteristics of MK-40 and a greater increase in the slope angle for the tryptophane-containing solution. This was associated with the intensification of electroconvection in the intensive current mode of electrodialysis near the less hydrophilic membrane surface.

The fluxes of heterocyclic amino acids having various side radical structures through the ion-exchange membranes during electrodialysis were mainly determined by the size factor and amino acid ion mobility. An increase in amino acid fluxes through the membranes operated with long-term electrodialysis, when not applying periodic membrane regeneration (fouled membranes), occurred in the overlimiting conditions, due to the conjugated transport of amino acid with water dissociation products; the effect of facilitated electromigration. Its influence increased with the increase in the catalytic activity of the membrane surface with respect to the water dissociation reaction. A decrease in the fluxes of the mineral salt ions with the increase in mass transfer of the amino acid in the intensive current mode was observed, due to the competitive transport of amino acid ions.

The performance of the electrochemical regeneration in the intensive current mode (the region of facilitated mass transport) showed that the organic fouling of the ion-exchange membranes in solutions of heterocyclic amino acids was reversible.

## Figures and Tables

**Table 1:** The properties of the studied heterocyclic amino acids.

Amino Acid	Structure	pI	pK		Molecular Weight	Solubility, g/100 mL H_2_O, 25 °C	Side Radical Volume, nm^3^
			pK_1_	pK_2_			
Tryptophan (Trp)	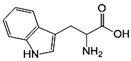	5.88	2.38	9.39	204.23	11.4	0.1755
Proline (Pro)	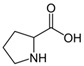	6.30	1.99	10.60	115.13	162.3	0.09

**Table 2:** Physical-chemical characteristics of the ion-exchange membranes used [44].

Membrane/Characteristic	MK-40	MA-41
Composite repeating unit	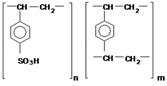	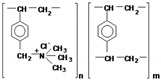
Thickness of wet membrane, microns	520 ± 10	450 ± 50
Exchange Capacity, mmol·g^−1^ wet	1.52 ± 0.08	1.18 ± 0.06
Water Content, g H_2_O/g wet, %	33 ± 1	35 ± 2
Transport number (0.5 M NaCl solution)	0.992	0.986
Density, g·cm^−3^ wet	1.19	1.14

**Table 3:** Roughness parameters of the ion-exchange membranes in different forms: H^+^-form of MK-40 and OH^−^-form of MA-41 (1), Pro-form (2), fouled membrane during electrodialysis of Pro containing solution (3), regenerated membrane (4).

Roughness Parameters	Membrane							
	MK-40 (1)	MK-40 (2)	MK-40 (3)	MK-40 (4)	MA-41 (1)	MA-41 (2)	MA-41 (3)	MA-41 (4)
Peak-to-peak, nm	2868.7	2868.6	2966.5	2869.0	2010.9	2079.7	2308.4	2020.2
Root mean square roughness, nm	485.3	528.6	761.6	490.7	161.1	170.4	208.9	162.2

## Data Availability

Not applicable.
